# The impact of socioenvironmental characteristics on domains of oral health-related quality of life in Brazilian schoolchildren

**DOI:** 10.1186/1472-6831-13-10

**Published:** 2013-01-28

**Authors:** Janice Simpson de Paula, Isabel Cristina Gonçalves Leite, Anderson Barbosa de Almeida, Glaucia Maria Bovi Ambrosano, Fábio Luiz Mialhe

**Affiliations:** 1 Department of Community Dentistry, Division of Health Education and Health Promotion, Piracicaba Dental School, University of Campinas –UNICAMP, P.O. BOX 52, Piracicaba, SP, 13414-903, Brazil; 2 Department of Community Dentistry, Division of Statistic, Piracicaba Dental School, University of Campinas –UNICAMP, P.O. BOX 52, Piracicaba, SP, 13414-903, Brazil; 3 Department of Public Health, Medicine School, Federal University of Juiz de Fora (UFJF), Juiz de Fora, MG, Brazil

## Abstract

**Background:**

Recent researches have pointed out the need to consider the functional and psychosocial dimensions of oral health, such as Oral Health-related Quality of Life (OHRQoL). The aim of this study was to investigate the influence of oral health status, socioeconomic factors and home environment of children on the four health domains of Child Perceptions Questionnaire (CPQ_11-14_).

**Methods:**

A cross-sectional study was conducted in Brazil with a sample of 286 schoolchildren aged 12 years and their parents. The schoolchildren were clinically examined, and participants were asked to complete the CPQ_11-14_, as well as a questionnaire about home environment. In addition, a questionnaire was sent to each child’s parents asking them about family socioeconomic status. The chi-square test and Poisson’s regression analysis were performed.

**Results:**

After adjusting for potential confounders, variables sex, monthly family income, mothers’ education showed a statistically significant association with all health domains of the CPQ_11-14_. The family structure and presence of bleeding impacted on emotional (p = 0.0135), and social (p = 0.0010) well-being health domain scores. Orthodontic treatment need showed a strong negative effect on functional limitations domain score (p = 0.0021).

**Conclusions:**

Clinical and socio-environmental factors had different impacts on domains of oral health-related quality of life, demonstrating the need to consider these conditions in planning strategies for the oral health of schoolchildren.

## Background

In recent decades, an exponential growth of scientific literature on quality of life (QoL) has been observed. In 1995, the World Health Organization extolled the quality of life concept as being “*individuals′ perception of their position in life in the context of the culture and value systems in which they live, and in relation to their goals, expectations, standards and concerns*” [1]. Thus, there is consensus that the quality of life of individuals is the result of social, economical, political and cultural aspects of society.

In accordance with this concept, in the field of oral health, the clinical criteria usually used by dentists to evaluate the patient’s oral health are problematic, because they do not always hold the same relevance for individuals’ functional or social needs [2]. This is corroborated by studies such as that of Agou et al. [3], which observed that although some patients presented severe occlusal problems, they rejected the need for orthodontic treatment for malocclusions perceived by dentists.

Therefore, contemporary concepts of health suggest that oral health should include not only normative assessment, but evaluation of physical functioning and psychosocial well-being as well [4,5]. According Petersen and Kwan [6], one barrier of the barriers to implementing community oral health promotion relates to the gap between training offered to oral health professionals and people’s perceived and real needs. For this reason, the association of quality of life with clinical conditions has been extensively studied [2-5,7-9].

Clinical indicators are important for the assessment of oral health and dental treatment needs, however their limitations must be considered in the planning of oral health interventions [5]. Thus, it is necessary to evaluate how dental problems and oral disorders interfere in the normal functioning of an individual’s life [10], as well as the importance of social determinants of dental health [11].

With this in mind, it is reasonable to assume that the relationship between Oral Heath-related Quality of Life (OHRQoL) and clinical conditions is also mediated by personal socio-environmental characteristics [12-16]. The OHRQoL is a multidimensional complex of interrelated domains, including absolute health and subjective perceptions [17-19]. These factors may have a strong influenced on multiple contexts and socioenvironmental characteristics, such as culture, economy, politics, education and family [19]. According to Wilson and Cleary [16] overall quality of life is determined by both medical and nonmedical factors, such as personal and environmental aspects. Full understanding of the other sources of impact oral diseases [20] and OHRQoL, such as social determinants of health [11,20], is important in order to begin the promotion of health actions.

In the case of children, Locker et al. [21] evaluated socioeconomic disparities in OHRQoL and found that income and family structure remained significant predictors of children’s OHRQoL, after controlling for oral disease variables. Moreover, as regards family environment, some studies have found that it plays a central role in the promotion of children’s oral health.

In general, the association between quality of life and oral health is measured by questionnaires properly validated for this purpose. The questionnaires are divided into domains, based on main dimensions of quality of life: functional limitations and well being [22]. Among the questionnaires about OHRQoL, there is the Child Perceptions Questionnaire (CPQ_11-14_), developed by a group of Canadian researchers, which evaluates the following subjective aspects: Oral Symptoms (OS), functional limitations (FL), emotional well-being (EWB), and social well-being (SWB) [9]. It is important to consider that each of these aspects may be influenced by different social determinants and clinical conditions. Therefore, evaluation by health domains (OS, FL, EWB, SWB) is a different way of analyzing these variables, involving a more detailed analysis of subjective perceptions about children’s OHRQoL.

The scientific literature presents some studies demonstrating associations between children’s oral health conditions and the socioeconomic factors that influence them, on the domains of OHRQoL instruments [3,4,8,9,12,21,23]. However, the impact of children’s home environment, such as family structure, number of siblings and household overcrowding on each domain of CPQ_11-14_ has not yet been sufficiently investigated.

The hypothesis of this study is that socioenvironmental aspects present different impacts on these domains of OHRQoL. The evaluation of this association is very important for oral health promotion planning, including intersectorial actions directed towards creating a healthy psychosocial environment for children.

### Purpose

The objective was to investigate the impact of variables related to children’s oral health status, socioeconomic factors and home environment on the four health domains of the Child Perceptions Questionnaire (CPQ_11-14._).

## Methods

### Ethical issues

The research project was submitted to the Ethics Committee of the Piracicaba Dental School, University of Campinas, Brazil, and approved under Protocol No. 055/2009.

### Study population

This cross-sectional study refers to a representative sample of children from Juiz de Fora, Brazil. Juiz de Fora is one of the pioneering cities in the industrial state of Minas Gerais, Brazil, and its predominating economic sectors are industry and services. The city has about 570,000 inhabitants, spread over a wide range of socio-economic backgrounds, of whom 98.91% have access to fluoridated water [24].

To calculate the probability sample, a 95% confidence interval level was adopted, 20% accuracy and design effect (deff) of 2. The sample size calculation was based on the DMFT (2.3) and standard deviation (2.72) of an epidemiological survey previously conducted [25]. The participants in the population-based study were 286 schoolchildren of both sexes, aged 12 years and their parents. The calculation to estimate the sample size was based on the effect of socioenvironmental and clinical characteristics of the OHRQoL, with association between CPQ11-14 scores and socioenvironmental and clinical covariates, considering 80% of power, confidence level of 5% and a prevalence ratio to be detected of at least 1.5. The minimum number of participants required by these parameters was 172.

Thus, 186 schoolchildren from 22 public and private schools were included in the conglomerate analysis, which was considered representative of the city, selected according a random multistage sampling design. First, schools were randomly selected, and in each school schoolchildren who fulfilled the inclusion criteria were included in the sample.

The study included all 12-year-old schoolchildren, whose parents consented to their participation in the research. Students with physical and/or cognitive impairments that would not allow the clinical examination and/or completion of the questionnaire were excluded from the study. In addition, schoolchildren with a history of previous or current orthodontic treatment were excluded.

### Clinical data

Data were collected by means of oral examination and self-administered questionnaires for schoolchildren and their parents. The schoolchildren were clinically evaluated at school by two calibrated examiners in an outdoor setting, under natural light, but not in direct sunlight. Community Periodontal Index (CPI) probes (ball-point) and mirrors were used according to the recommendations of the World Health Organization for epidemiological surveys [26]. An orthodontic specialist (ABA) and a Community Dental Health specialist (JSP), both with wide experience in epidemiological surveys, conducted the survey data collection. Prior to the survey, there was a calibration phase for all clinical variables with practical and theoretical activities at intermittent periods totaling 24 h, performed by a gold standard examiner. Good intra-examiner reproducibility (Kappa > 0.91) was reached.

One of the authors (JSP) assessed the presence of decayed, missing, and filled teeth in the permanent and primary dentition (DMFT and dmft index). For statistical analysis, the presence or absence of untreated caries was evaluated according to the D component of DMFT index. Dental trauma (presence/absence), enamel defects (Developmental Defects of Enamel index – presence/absence), periodontal status (presence/absence of bleeding) and dental treatment needs (yes/no) were also evaluated in accordance with WHO criteria [26].

In addition, the schoolchildren were classified for caries risk in accordance with American Academy of Pediatric Dentistry (AAPD) criterion: high risk (urgency treatment need, abscesses, presence of active white spots, cavitated caries lesions), moderate risk (presence of restored teeth, chronic caries or inactive white spot lesion), and low risk (sound teeth, absence of white spot lesion and cavitated caries lesion) [27].

The difference between the active or non active lesions was evaluated according to the criteria of Nyvad et al. [28] when active lesions were cavitated and had softened tissue. Furthermore, initial/white spot lesions were defined as opacities of white, or brownish color, oval or round shaped, following the gingival margin with decreased enamel translucency and clearly defined from the adjacent enamel [28].

Another examiner (ABA) collected the Malocclusion data according the Dental Aesthetic Index (DAI). The DAI measures the social acceptability of a child’s dental appearance, based on collecting and attributing weight to 10 occlusal traits. The DAI score ranges from 13 (the most socially acceptable) to 100 (the least acceptable), and orthodontic treatment needs can be prioritized on the basis of predefined categories: having more acceptable dental appearance (score DAI < 31 – no orthodontic treatment need) or having less acceptable dental appearance (score DAI ≥ 31 – orthodontic treatment need) [29].

### Home environment characteristics

The participants were asked to answer the questions about home environment: structure family (children live with both biological parents – yes/no), number of siblings (<2 and 2 or more), and household overcrowding: number of people living in the household per number of rooms (≤ 1 person per room or > 1 person per room). Data were collected on sex and type of school. These variables were selected, collected and categorized based on the study of Paula et al. [30].

### Oral health-related quality of Life (OHRQoL) data

After this, the Child Perceptions Questionnaire (CPQ_11-14_), developed by Jokovic et al. [9] was applied. The Brazilian version of this questionnaire was translated and validated by Barbosa et al. [4], and presents good psychometric properties. The CPQ_11-14_ instrument may be self- administered or interviewer-administered with small difference in results of scores [31], and for this study we applied it in the self-administered form. The CPQ_11-14_ determine quality of life associated with oral health and consists of 36 items, grouped into 4 health domains: oral symptoms (OS) – 6 questions, functional limitations (FL) – 9 questions, emotional well-being (EWB) – 9 questions, and social well-being (SWB) – 12 questions. In each item questions are asked about the frequency of events, as applied to the teeth, lips, and jaws, in the last 3 months. The responses are given using a Likert-type scale based on the number of points in the scale: “Never” = 0; “Once or twice” = 1; “Sometimes” = 2; “Often” = 3; and “Very often” = 4. Although the instrument is designed to yield an overall score, a separate score can be generated for each of the four domains. Higher scores signify worse OHRQoL.

### Socioeconomic characteristics

A questionnaire was sent to children’s parents asking about the family socioeconomic status. Data about monthly family income, parents’ educational level and home ownership were collected. The monthly family income was measured based on the number of minimum wages which the family receives (up to 3/4 or more), considering the Brazilian minimum wage at time of data collection of approximately US $ 290 per month. The parents’ educational level was categorized by number of years of schooling into two levels: up to 8 years of schooling or over 8 years.

These variables were selected, collected and categorized based on study of Paula et al. [30] about socioeconomic characteristics.

### Statistical analyses

Data were analyzed using descriptive statistics, bivariate analysis, and a regression model. Aggregated health domain CPQ_11-14_ scores were calculated and dichotomized by the median (dependent variable). The chi square test was used for comparisons between proportions. For health domain scores, CPQ_11-14_ scores were calculated for the prevalence ratio with regard to socio-environmental and clinical factors.

A Poisson regression model was used to assess the association between the predictor variables and outcomes. It is considered an appropriate model to determine associations between dependent and independent variables, and allows control of confounding factors. A backward stepwise procedure for levels was used to include or exclude explanatory variables in the adjustments for the models, based on the conceptual framework of the hierarchical approach [32] considering distal (demographic), medial (socioenvironmental) and proximal (clinical conditions) characteristics. Explanatory variables presenting a p value ≤ 0.20 in the assessment of association with each outcome (bivariate analyses) were included in the adjustments for the model. Variables that were not related and did not contribute significantly to the model were eliminated and the final model contained only factors that remained associated at the level p ≤ 0.05. The statistical tests were performed using the SAS software [33].

## Results

Of the participants, 203 (71%) were from public school and 164 (57.3%) were girls. The mean DMFT index value was 1.12 (SD 1.75) and dmft was 0.49 (SD 0.82). The prevalence of trauma was 2.6% (8 participants), and enamel defects 28.3% (52 participants). It was observed that 67 (23.4%) of participants had no dental treatment needs.

For caries risk, there were 147 (51.4%) with low, 47 (16.4%) moderate, and 92 (32.2%) children with high risk. The presence of bleeding was observed in 14.7% (42) of the schoolchildren. DAI scores ranged from 6.24 to 56.46 with a mean of 26.51 (SD 6.24), and 78 (27.3%) needed orthodontic treatment (DAI ≥ 31). The mean overall CPQ _11–14_ score was 24.08 (SD 21.95), ranging from 0 to 106. The median for overall CPQ _11–14_ was 18; for the OS domain it was 5; FL domain, 3; EWB domain, 5; and for the SWB domain it was 2.5.

Table 1 presents the socioeconomic status variables that showed significant association with a score above the median in the CPQ domain scores_11-14_. Children at public schools, with family incomes of up to 3 minimum wages, and mothers with up to 8 years of education presented significant association with all domains (OS, FL, EWB and SWB).

**Table 1:** **Bivariate analysis of association between socioeconomic status with oral health- related quality of life in the domains median scores of CPQ**_**11-14**_**(n = 286)**

**Variables**	**N**	**Oral symptoms**			**Functional limitations**			**Emotional well-being**			**Social well-being**		
		**Scores > median n (%)**	**PR**^**1**^**crude**	**P**	**Scores > median n (%)**	**PR crude**	**p**	**Scores > median n (%)**	**PR crude**	**p**	**Scores > median n (%)**	**PR crude**	**P**
Sex													
*Boy*	122	44 (36.07%)	1.00		49 (40.16%)	1.00		49 (40.16%)	1.00		53 (43.44%)	1.00	
*Girl*	164	90 (54.88%)	1.52	0.0016	92 (56.10%)	1.40	0.0077	90 (54.88%)	1.37	0.0138	90 (54.88%)	1.26	0.0558
School type													
*Private*	83	18 (21.69%)	1.00		20 (24.10%)	1.00		16 (19.28%)	1.00		15 (18.07%)	1.00	
*Public*	203	116 (57.14%)	2.63	<0.0001	121 (59.61%)	2.47	<0.0001	123 (60.59%)	3.14	<0.0001	128 (63.05%)	3.49	<0.0001
Family income													
*> 3 wages*^*2*^	72	16 (22.22%)	1.00		20 (27.78%)	1.00		14 (19.44%)	1.00		13 (18.06%)	1.00	
*≤ 3 wages*	214	118 (55.14%)	2.48	<0.0001	121 (56.54%)	2.04	<0.0001	125 (58.41%)	3.00	<0.0001	130 (60.75%)	3.36	<0.0001
Father’s education													
*≤ 8 years*	124	67 (54.03%)	1.00		67 (54.03%)	1.00		71 (57.26%)	1.00		75 (60.48%)	1.00	
*> 8 years*	108	38 (35.19%)	0.65	0.0040	42 (38.89%)	0.72	0.0212	39 (36.11%)	0.67	0.0013	35 (32.41%)	0.54	<0.0001
Mother’s education													
*≤ 8 years*	141	83 (58.87%)	1.00		86 (60.99%)	1.00		86 (60.99%)	1.00		90 (63.83%)	1.00	
*> 8 years*	142	48 (33.80%)	0.57	<0.0001	53 (37.32%)	0.61	<0.0001	50 (35.21%)	0.60	<0.0001	51 (35.92%)	0.56	<0.0001
Home ownership													
*Yes*	156	65 (41.67%)	1.00		69 (44.23%)	1.00		70 (44.87%)	1.00		67 (42.95%)	1.00	
*No*	130	69 (53.08%)	1.27	0.0542	72 (55.38%)	1.25	0.0603	69 (53.08%)	1.17	0.1668	76 (58.46%)	1.36	0.009

^1^*PR*, Prevalence Ratio ^2^ Brazilian minimum wage in effect at time of data collection = US$ 290.

Table 2 shows the prevalence ratios of home environmental and CPQ_11-14_ scores. Children not living with both biological parents and living with 2 or more siblings showed statistically significant associations with negative impact on OHRQoL for all CPQ domains_11-14_. The clinical variables DMFT and their components trauma, enamel defects were excluded from Table 2, because they were not statistically significant, with dependent variables.

**Table 2:** **Bivariate analysis of home environment variables with oral health- related quality of life in the domains median scores of CPQ**_**11-14**_**(n = 286)**

**Variables**	**N**	**Oral symptoms**			**Functional limitations**			**Emotional well-being**			**Social well-being**		
		**Scores > median n (%)**	**PR crude**	**p**	**Scores > median n (%)**	**PR crude**	**p**	**Scores > median n (%)**	**PR crude**	**p**	**Scores > median n (%)**	**PR crude**	**p**
Children live with													
*Both biological parents*	161	63 (39.13%)	1.00		67 (41.61%)	1.00		65 (40.37%)	1.00		67 (41.61%)	1.00	
*Others*	125	71 (56.80%)	1.45	0.0030	74 (59.20%)	1.42	0.0032	74 (59.20%)	1.46	0.0016	76 (60.80%)	1.46	0.0013
Siblings													
*No*	58	20 (34.48%)	1.00		22 (37.93%)	1.00		22 (37.93%)	1.00		25 (43.10%)	1.00	
*Yes*	228	114 (50.00%)	1.45	0.0345	119 (52.19%)	1.38	0.0524	117 (51.32%)	1.35	0.0686	118 (51.75%)	1.20	0.2394
Number of siblings													
*< 2*	143	54 (37.76%)	1.00		54 (37.76%)	1.00		57 (39.84%)	1.00		58 (40.56%)	1.00	
*2 or more*	143	80 (55.94%)	1.48	0.0021	87 (60.84%)	1.61	<0.0001	82 (57.34%)	0.70	0.0031	85 (59.44%)	1.47	0.0014
Household overcrowding													
*≤ 1 person/room*	241	106 (43.98%)	1.00		117 (48.55%)	1.00		113 (46.89%)	1.00		114 (47.30%)	1.00	
*More than 1 person/room*	45	28 (62.22%)	1.41	0.0244	24 (53.33%)	1.10	0.5556	26 (57.78%)	1.23	0.1797	29 (64.44%)	1.36	0.0348

Table 3 presents associations observed between clinical conditions and OHRQoL for domains. There was a strong association between the presence of bleeding and orthodontic treatment need with OS, FL, EWB and SWB domains.

**Table 3:** **Bivariate analysis of clinic condition variables with oral health- related quality of life in the domains median of CPQ**_**11-14**_**(n = 286)**

**Variables**	**n**	**Oral symptoms**			**Functional limitations**			**Emotional well-being**			**Social well-being**		
		**Scores > median n (%)**	**PR crude**	**p**	**Scores > median n (%)**	**PR crude**	**p**	**Scores > median n (%)**	**PR crude**	**p**	**Scores > median n (%)**	**PR crude**	**p**
D (caries)													
*absence*	234	106 (45.30%)	1.00		111 (47.44%)	1.00		108 (46.15%)	1.00		109 (46.58%)	1.00	
*Presence*	52	28 (53.85%)	1.19	0.2632	30 (57.69%)	1.22	0.1809	31 (59.62%)	1.29	0.0790	34 (65.38%)	1.40	0.0142
DMFT													
> 0	175	76 (43.43%)	1.00		84 (48.00%)	1.00		79 (45.14%)	1.00		78 (44.57%)	1.00	
= 0	111	58 (52.25%)	1.20	0.1450	57 (51.35%)	1.07	0.5806	60 (54.05%)	1.20	0.1417	65 (58.56%)	1.31	0.0211
Dental treatment need													
*No*	219	97 (44.29%)	1.00		104 (47.49%)	1.00		98 (44.75%)	1.00		99 (45.21%)	1.00	
*Yes*	67	37 (55.22%)	1.25	0.1166	37 (55.22%)	1.16	0.2678	41 (61.19%)	1.37	0.0184	44 (65.67%)	1.45	0.0034
Caries risk													
*Low*	147	63 (42.86%)	1.00	0.2115	69 (46.94%)	1.00	0.7081	59 (40.14%)	1.00	0.0045	63 (42.86%)	1.00	0.0043
*Moderate*	47	21 (44.68%)	1.04		24 (51.06%)	1.09		23 (48.94%)	1.22		21 (44.68%)	1.04	
*High*	92	50 (54.37%)	1.27		48 (52.17%)	1.11		57 (61.96%)	1.54		59 (64.13%)	1.50	
Bleeding													
*No*	244	109 (44.67%)	1.00		114 (46.72%)	1.00		109 (44.67%)	1.00		110 (45.08%)	1.00	
*Yes*	42	25 (59.52%)	1.33	0.0748	27 (64.29%)	1.38	0.0355	30 (71.43%)	1.60	0.0014	33 (78.57%)	1.74	<0.0001
Orthodontic treatment need													
*No*	208	89 (42.79%)	1.00		91 (43.75%)	1.00		93 (44.71%)	1.00		94 (45.19%)	1.00	
*Yes*	78	45 (57.69%)	1.35	0.0245	50 (64.10%)	1.47	0.0022	46 (58.97%)	1.32	0.0316	49 (62.82%)	1.39	0.0079

Table 4 summarizes the results of the regression model and the total amount of variance, in domain CPQ_11-14_ scores. Statistically significant variables were included in the Poisson regression model. After adjusting for potential confounders, it was found that the variables sex, monthly family income, mothers’ education showed a statistically significant association with all health domains. In addition, the family structure (children no live with both biological parents) and presence of bleeding impacted on emotional and social well-being health domain scores. The orthodontic treatment need showed a strong negative effect on the functional limitation domain score.

**Table 4:** **Association between socio-environmental and clinical condition variables with oral health- related quality of life in the domains median score of CPQ**_**11-14**_**, through the Poisson model for multiple regression analysis**

**Variables**	**Oral symptoms**			**Functional limitations**			**Emotional well-being**			**Social well-being**		
	**RP**_**adj**_^**1**^	**CI**^**2**^**- 95%**	**p**	**RP**_**adj**_^**1**^	**CI - 95%**	**p**	**RP**_**adj**_^**1**^	**CI - 95%**	**p**	**RP**_**adj**_^**1**^	**CI - 95%**	**p**
Sex												
Boy	1.00			1.00			1.00			1.00		
Girl	1.13	1.05-1.22	0.0013	1.10	1.02-1.9	0.0105	1.10	1.02-1.18	0.0113	1.08	1.01-1.16	0.0363
Family income												
> 3 minimum wages	1.00			1.00			1.00			1.00		
≤ 3 minimum wages	1.20	1.08-1.32	0.0002	1.15	1.04-1.27	0.0076	1.21	1.10-1.34	0.0002	1.24	1.13-1.37	<0.0001
Mother’s education												
≤ 8 years	1.00			1.00			1.00			1.00		
> 8 years	0.90	0.83-0.98	<0.0001	0.90	0.83-0.98	0.0177	0.92	0.84-0.99	0.0401	0.92	0.85-0.99	0.0315
Children live with												
both biological parents							1.00			1.00		
Others		-			-		1.08	1.01-1.16	0.0352	1.08	1.01-1.16	0.0243
Bleeding												
No							1.00			1.00		
Yes		-			-		1.13	1.02-1.24	0.0135	1.16	1.06-1.27	0.0010
Orthodontic treatment need												
No				1.00								
Yes		-		1.13	1.04-1.22	0.0021		-			-	

^1^ Prevalence Ratio Adjusted ^2^ Confidence interval.

## Discussion

This cross-sectional study evaluated a population-based sample of 12-year-old schoolchildren and found that socio-environmental factors had negative impact on the health domains of CPQ_11-14_ . The statistical regression model used, allowed the confounding variables to be controlled and to determine the influence of these variables on oral symptoms, functional limitations, emotional well-being, and social well-being health domains.

Gender was associated with all domains of OHRQoL, as has been found in other studies [2,13,15]. This impact may be related to self-esteem aspects and self-perception about oral health and body image [2,3,34]. Honkala et al. [35] confirmed the significant association between female and frequency in toothbrushing, and this question is related with higher socioeconomic status of the family and self-esteem. Foster Page et al. [34] highlights the contribution of psychosocial characteristics of gender to OHRQoL perception.

According to Locker et al. [13], socioeconomic status presented associations with OHRQoL when overall CPQ_11-14_ scores were evaluated. Nevertheless, up to now only Piovesan et al. [15] analyzed the impact of these variables on each of the health domains of CPQ_11-14_, in a similar way to the approach used in the present study. The results of this investigation found that monthly family income and mother’s education level had strong impact on oral health-related quality of life, observed in those of each of the four health domains of the CPQ_11-14_.

The home environment, especially family structure (children not living with both biological parents), was also associated with OHRQoL. Furthermore, it was found that this variable had a negative impact on the EWB and SWB domains of CPQ_11-14_. These data have not yet been investigated in other studies, but it can be hypothesized that this relationship could be due to the influence of home environment on schoolchildren’s oral health behavior [36-38].

Orthodontic treatment need showed a strong negative effect on the FL domain score of CPQ_11-14_. The functional limitations domain evaluated the following aspects: breathing through the mouth, taking longer than others to eat a meal, have trouble sleeping, find it difficult to bite or chew food, such as apples, corn on the cob or steak, difficulty in opening the mouth wide, difficult to say some words, difficult to eat foods one would like to eat, difficult to drink through a straw, difficult to drink or eat hot or cold foods [2,3]. These results differed from those of other studies published in the literature, which observed that malocclusion showed a negative effect on the emotional and social well-being domain scores in children [2,23]. The present study verified that the impact orthodontic treatment need has on OHRQoL is related not only to correcting dental aesthetics, as affirmed by O’Brien et al. [2], but is also to the perception of functional limitations.

There was a statistically significant association of the presence of bleeding with emotional well-being (feelings about yourself and what others think about you) and social well-being (difficulty at school, in activities, avoiding smiling, difficulties in relationships with other children) domains of the CPQ_11-14_. The results were similar to those of Lopez and Baelum [39] who found association between periodontal diseases and oral health-related quality of life. However, the authors used a different OHRQoL instrument (Oral Health Impact Profile - OHIP) and did not evaluate the impact of periodontal diseases on the OHRQoL instrument by its domains.

According to the descriptive data, the prevalence of dental caries in the population of this study was small, with 234 (81.8%) schoolchildren presented absence of caries and 111 (59.6%) with DMFT equal to zero. This aspect could explain the low association founded between the presence of caries and the domains oral symptoms and functional limitations of CPQ_11-14_. The aesthetic questions that interfere in adolescents’ social relationships was the domain of CPQ_11-14_ most influenced by oral health problems, corroborating the results found in this population by Agou et al. [3].

Marmot and Bell emphasized areas for action in Social Determinants of Health, including improving the individual conditions and structural drivers. Therefore, in this study it was demonstrated that not only clinical conditions are associated with OHRQoL, but social and environmental variables interfere significantly in children’s conditions of daily life [40,41]. Therefore, health promotion should be based on awareness of environmental factors [6], and health policies should be reoriented to incorporate oral health using socio-dental approaches.

The data of this research should be interpreted within the context of some limitations. The cross-sectional design of this study made it difficult to evaluate the risk indicators of OHRQoL. The measures of behavior and self-esteem, which might influence the oral health conditions and subjective perception of the schoolchildren, were not included. Therefore, longitudinal studies are necessary for better determination of the associations between confounding variables that could have an influence on OHRQoL.

## Conclusion

In conclusion, it was found that some clinical, socioeconomic and home environment factors had different impacts on domains of oral health-related quality of life, demonstrating the need to consider these conditions when planning oral health strategies applicable to schoolchildren.

## Competing interests

The authors declare that they have no competing interests.

## Authors’ contributions

JSP participated in the conception and design of the study, data interpretation, data acquisition, and drafting the manuscript. ICGL contributed to the conception and design of the study. ABA contributed to the data collection. GMBA participated in data analyses. ACP contributed to critical revision of manuscript. FLM participated in the conception and design of the study and critical revision of manuscript. All authors read and approved the final manuscript.

## Pre-publication history

The pre-publication history for this paper can be accessed here:

http://www.biomedcentral.com/1472-6831/13/10/prepub
